# Exploring the Relationship Between Lipoprotein (a) Level and Myocardial Infarction Risk: An Observational Study

**DOI:** 10.3390/medicina60111878

**Published:** 2024-11-16

**Authors:** Ionut Cezar Buciu, Eugen Nicolae Tieranu, Andreea Stefania Pircalabu, Octavian Istratoaie, Ovidiu Mircea Zlatian, Ramona Cioboata, Ionut Donoiu, Constantin Militaru, Sebastian Militaru, Cristian Militaru

**Affiliations:** 1 Doctoral School, University of Medicine and Pharmacy of Craiova, 200349 Craiova, Romania; cezarbuciu@yahoo.com; 2 Department of Cardiology, University of Medicine and Pharmacy of Craiova, 200349 Craiova, Romania; eugen.tieranu@umfcv.ro (E.N.T.); octavian.istratoaie@umfcv.ro (O.I.); ionut.donoiu@umfcv.ro (I.D.); constantin.militaru@umfcv.ro (C.M.); crmilitaru@gmail.com (C.M.); 3 Department of Cardiology, Craiova Emergency Clinical County Hospital, 200642 Craiova, Romania; 4 Department of Oncology, Craiova Emergency Clinical County Hospital, 200642 Craiova, Romania; pircalabuandreea@yahoo.com; 5 Department of Microbiology, University of Medicine and Pharmacy of Craiova, 200349 Craiova, Romania; ovidiu.zlatian@umfcv.ro; 6 Medical Laboratory, Craiova Emergency Clinical County Hospital, 200642 Craiova, Romania; 7 Pneumology Department, University of Medicine and Pharmacy, 200349 Craiova, Romania; ramona_cioboata@yahoo.com; 8 Pneumology Department, Victor Babes University Hospital Craiova, 200515 Craiova, Romania; 9 Cardiomed Hospital, 200032 Craiova, Romania

**Keywords:** Lipoprotein (a), acute myocardial infarction, young patients, risk factors, cardiovascular disease

## Abstract

*Background and Objectives*: This observational study investigates the relationship between Lipoprotein (a) (Lp(a)) levels and the risk of acute myocardial infarction (AMI). This study aims to highlight the association of elevated Lipoprotein (a) levels with an increased atherogenic profile and the potential risk of AMI. *Materials and Methods*: We conducted a case–control study involving 106 individuals, including 64 AMI patients (both STEMI and NSTEMI) and 42 healthy controls. Comprehensive clinical and biochemical assessments, including Lp(a) measurements, were conducted. *Results*: Patients with Lp(a) levels ≥ 30 mg/dL had a threefold increased risk of AMI compared to those with lower levels, independent of traditional risk factors such as cholesterol, smoking, and body weight. Elevated Lp(a) was observed in 50% of AMI patients compared to 28.57% in controls (*p* = 0.028). Notably, a multivariate analysis identified high Lp(a) levels, low HDL-C levels, and obesity as significant independent predictors of AMI, indicating these factors may contribute to AMI risk more prominently than other conventional risk factors in patients with elevated Lp(a). Moreover, the association between Lp(a) and AMI risk was consistent across various patient subgroups, with low HDL-C further compounding the risk. *Conclusions*: Lp(a) is a significant independent risk factor for acute myocardial infarction; therefore, screening for Lp(a) levels can help identify high-risk individuals beyond traditional markers. Therapeutic approaches targeting Lp(a) may reduce AMI incidence. Future research should explore how Lp(a) promotes atherosclerosis and assess Lp(a)-lowering therapies to improve patient outcomes.

## 1. Introduction

Acute myocardial infarction (AMI) continues to be a burden to the global healthcare system and remains one of the leading causes of mortality and morbidity [1]. With economic development and the rise in global living standards, humanity faces an increase in the prevalence of major risk factors, such as obesity, diabetes, hypertension, and dyslipidemia, which contribute to the percentage of global mortality causality. Traditionally, risk factors such as smoking, dyslipidemia, and high blood pressure have been at the core of prevention and treatment strategies [2]. However, recent attention has shifted toward the role of Lipoprotein (a) in the pathogenesis of cardiovascular diseases, particularly in its contribution to the risk of myocardial infarction (MI) [3].

Lipoprotein (a) is an emerging biomarker, relatively new, known for its atherogenic and pro-thrombotic properties. Lp(a) is a particle in the blood that carries cholesterol. High levels of Lp(a) have been implicated in the development of atherosclerosis and other cardiovascular conditions, making it a potentially significant predictor of MI. Moreover, while Lp(a) is generally considered stable over an individual’s lifetime due to its strong genetic basis, this study revealed that Lp(a) levels may increase following an MI, potentially as part of an inflammatory response. Such findings emphasize that measuring Lp(a) solely in the peri-infarct period might not fully reflect the biomarker’s role in assessing long-term cardiovascular risk, suggesting the value of repeated Lp(a) assessments in post-MI patients for more accurate risk stratification [4]. Additionally, integrating Lp(a) into routine cardiovascular assessments could help identify high-risk patients with otherwise normal LDL-C levels, offering a more comprehensive risk profile [5]. Despite these associations, the specific mechanisms through which Lp(a) contributes to MI and its interaction with other risk factors such as obesity, diabetes, and hypertension remain unclear [6]. Lp(a) is a particle in the blood that carries cholesterol [4].

This study aimed to address this knowledge gap by examining the role of Lp(a) in the pathogenesis of MI.

## 2. Materials and Methods

The study population consisted of 106 individuals of similar age, of whom 64 were diagnosed with acute myocardial infarction and 42 were included in the control group. Patients with myocardial infarction were admitted to the Cardiology Clinic of the Emergency County Hospital of Craiova and diagnosed with myocardial infarction in either STEMI or NSTEMI presentation. All patients with myocardial infarction underwent coronary angiography and angioplasty procedures and were diagnosed with coronary artery disease. Exclusion criteria were the absence of coronary lesions, familial hypercholesterolemia, age > 55 years, age < 18 years, and in-hospital death.

The control group comprised 42 healthy individuals who underwent routine check-ups at the outpatient clinic of the Emergency County Hospital of Craiova. The patients were clinically and paraclinically evaluated (through electrocardiography and echocardiography). The same exclusion criteria were applied, along with the absence of high blood pressure and diabetes.

All patients provided written consent and were subsequently subjected to detailed clinical and paraclinical evaluations and underwent 12-lead electrocardiograms using the MAC2000 system (GE Healthcare, New York, NY, USA) and transthoracic echocardiograms with the Vivid E90 device (GE Healthcare, Chicago, IL, USA). Biological tests were performed in both groups at the hospital laboratory in Craiova. 

**Figure 1:**
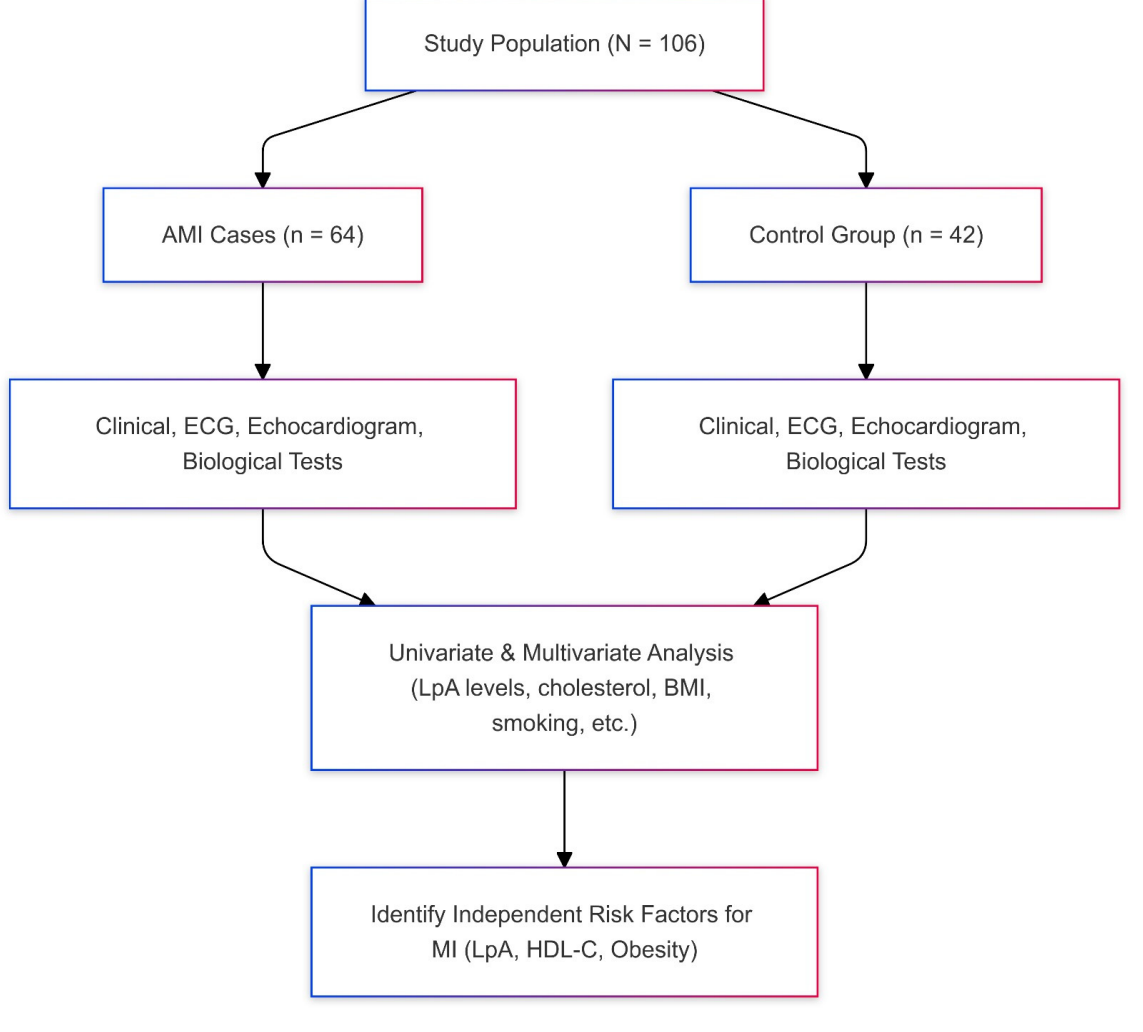
Study design and workflow of an observational case–control study investigating the relationship between Lipoprotein (a) (Lp(a)) levels and the risk of acute myocardial infarction (AMI).

The study design (Figure 1) was structured to ensure that both groups underwent comprehensive clinical assessments including ECG, echocardiograms, and biological tests. Univariate and multivariate analyses were performed to identify independent risk factors for AMI, focusing on Lp(a), HDL-C, and obesity. The results showed that elevated Lp(a) levels (≥30 mg/dL) [7] were significantly linked to a threefold increase in AMI risk, independent of traditional risk factors such as cholesterol levels, smoking, and body weight. Multivariate analysis further identified high Lp(a) levels, low HDL-C levels, and obesity as independent predictors of AMI, suggesting that these factors may play a more prominent role in AMI risk than conventional risk factors in patients with elevated Lp(a).

*Collection of blood samples*. Samples were collected from all participants under fasting conditions to ensure accurate biochemical assessment. Samples were collected early in the morning following an overnight fast of at least 12 h. Venous blood was drawn into appropriate collection tubes, and the samples were immediately processed to separate the plasma. Plasma was obtained by centrifugation at 1500× *g* for 10 min at room temperature and then refrigerated until further analysis.

*The biological parameters measured* included complete blood count and total cholesterol, HDL-C, LDL-C, and Lipoprotein (a) levels. Blood counts were determined using an Alinity-H analyzer (Abbott Diagnostics, Abbott Park, IL, USA), while blood chemistry, including Lp(a), was determined using the COBAS INTEGRA 400/700/800 system (Roche Diagnostics, Basel, Switzerland). 

Lp(a) levels were determined using the Tina-quant Lipoprotein (a) (Latex) assay, which is a particle-enhanced immunoturbidimetric assay. In this method, latex particles coated with antibodies specific to Lp(a) were used to precipitate Lp(a) present in the sample. The precipitate was then measured turbidimetrically at a 552 nm wavelength. This method was designed for the quantitative immunological determination of Lp(a) in human serum and plasma, ensuring a high degree of specificity and reproducibility. The within-run and between-run coefficients of variation were maintained below 2%, ensuring a high precision for Lp(a) measurements.

*Statistical analysis* was performed using the STATA 17 SE software (StataCorp LLC, College Station, TX, USA). The statistical tests used included univariate analysis to compare Lipoprotein (a) levels and other risk factors between the myocardial infarction and control groups. Statistical tests such as the chi-square test for categorical variables (e.g., smoking and sex) and the Mann–Whitney test for continuous variables (e.g., cholesterol levels) were applied.

Logistic regression was used to evaluate the independent contribution of elevated Lipoprotein (a) levels (≥30 mg/dL) to the risk of myocardial infarction, adjusting for other risk factors (e.g., HDL, LDL, sex, and obesity). This stratification has been reported in the existing literature, which demonstrates that Lipoprotein (a) concentrations ≥ 30 mg/dL are associated with a significantly elevated risk of myocardial infarction (Cai et al., 2019) [5]. This particular breakpoint was chosen after a literature review showed that most studies used this value of Lp(a) as a cut-off. A group comparison analysis indicated significant differences between myocardial infarction cases and controls concerning cholesterol levels, weight status, and smoking proportion. In the multivariate logistic regression analysis, traditional risk factors such as total cholesterol, HDL, LDL, smoking, and gender were controlled for, allowing the identification of Lipoprotein (a) as an independent risk factor for myocardial infarction.

A *p*-value < 0.05 was considered statistically significant for all tests, and data analysis followed the intention-to-treat principle to assess whether Lipoprotein (a) levels are an independent predictor of myocardial infarction (MI) risk and to explore how other risk factors, such as obesity, smoking, cholesterol, and gender, influence this relationship.

## 3. Results

The study results are presented in Table 1, and demonstrate statistically significant differences between the control and acute myocardial infarction (AMI) groups across several key health parameters. Although the AMI group exhibited a higher proportion of male participants (79.69%) compared to the control group (66.67%), this difference did not reach statistical significance (*p* = 0.132). Body weight disparities were highly significant (*p* < 0.001), with the MI group displaying elevated rates of overweight (34.38%) and obesity (39.06%) relative to the control group. The MI group had a greater proportion of smokers (68.75%) compared to the control group (50.00%), with a borderline significant difference (*p* = 0.050).

Cholesterol profiles were significantly worse in the MI group. They had higher total cholesterol (median 205 mg/dL) compared to the control group (median 172 mg/dL, *p* = 0.002), higher LDL cholesterol levels (*p* = 0.006), and lower HDL cholesterol levels (*p* < 0.001). 

The study’s findings on full blood count (FBC), left ventricular ejection fraction (LVEF), and cardiac biomarkers CK-MB and troponin offer a detailed view of the physiological disruptions associated with myocardial infarction (MI). Patients in the MI group showed a significantly elevated white blood cell (WBC) count, with a median of 8.9 × 10^3^/μ/L compared to 7.0 × 10^3^/μ/L in the control group, suggesting an inflammatory response to the cardiac injury. While other FBC components like hemoglobin and platelet counts did not vary significantly between groups, this increase in WBCs highlights inflammation as a critical component of MI pathophysiology. Cardiac function, as reflected in LVEF, was also notably compromised in MI patients, who showed a median LVEF of 50%, lower than the control group’s 61%, indicating weakened ventricular performance following myocardial damage. The cardiac biomarkers CK-MB and troponin, known for their specificity to cardiac injury, showed stark elevations in the MI group, with CK-MB reaching a median level of 310 compared to 7.6 in controls, and troponin levels at 3.1 ng/L versus 0.7 ng/L, confirming extensive myocardial cell damage. Together, these findings underscore the inflammatory, structural, and biochemical impacts of myocardial infarction, with inflammation, reduced cardiac output, and cardiac muscle damage emerging as core elements of the acute response to MI.

Elevated Lipoprotein (a) levels (≥30 mg/dL) were observed with greater frequency in the MI group (50%) compared to the control group (28.57%), demonstrating a statistically significant difference (*p* = 0.028).

The distribution of Lipoprotein (a) levels among control subjects and patients with myocardial infarction (MI) across different age groups showed that in individuals under 40 years old, MI patients exhibit markedly higher median Lp(a) levels compared to controls, while the control group showed a higher median Lp(a) level than the MI patients in the 40–50 years age bracket, and in individuals over 50 years old, both groups had relatively low and comparable median Lp(a) levels.

Lipoprotein (a) levels varied significantly. For individuals with a standard weight, the MI group exhibited considerably higher median Lp(a) levels (82.1 mg/dL) compared to the control group (12.9 mg/dL). This suggests that among those with a standard weight, elevated Lp(a) levels may be associated with an increased risk of myocardial infarction. The notable difference highlights Lp(a) as a potential independent risk factor for cardiovascular events, even in individuals without excess body weight.

In contrast, the trend shifts within the overweight and obese categories. Among overweight individuals, the MI group had a slightly lower median Lp(a) level (19.35 mg/dL) than the control group (27.1 mg/dL). For obese participants, the control group recorded a much higher median Lp(a) level (89.2 mg/dL) than the MI group (23.9 mg/dL). These findings suggest that in individuals with higher body mass, the relationship between Lp(a) levels and MI risk may be less direct, potentially due to confounding factors linked to obesity, such as metabolic disturbances or other lipid abnormalities.

The distribution of Lipoprotein (a) (Lp(a)) levels across age and weight categories revealed distinct patterns in the myocardial infarction (MI) and control groups. Among the individuals younger than 40 years, MI patients exhibited markedly higher median Lp(a) levels compared to controls, suggesting that elevated Lp(a) may be particularly influential in this younger cohort. However, this trend shifted in individuals aged 40 to 50, where control participants unexpectedly presented with higher median Lp(a) levels than MI patients, while in those over 50, both groups showed relatively comparable and lower Lp(a) levels. When examining Lp(a) distribution across weight categories, standard-weight individuals with MI had significantly elevated median Lp(a) levels (82.1 mg/dL) compared to their control counterparts (12.9 mg/dL). Conversely, in the overweight and obese categories, the MI group showed lower median Lp(a) levels than the control group, with obese controls exhibiting notably higher Lp(a) levels (89.2 mg/dL) than their MI counterparts (23.9 mg/dL). These variations suggest that Lp(a) may play a differential role in cardiovascular risk depending on age and weight status.

Univariate analysis of the risk of acute myocardial infarction in patients with high Lipoprotein (a) levels.

The data show distinct patterns when comparing individuals with low (<30 mg/dL) (Table 2) versus high (≥30 mg/dL) (Table 3) levels of Lipoprotein (a) in both the control and myocardial infarction (MI) groups.

Low Lipoprotein (a) (<30 mg/dL) (Table 2):

In individuals with low Lipoprotein (a), significant differences were observed between the myocardial infarction (MI) and control groups. The MI group had a much lower percentage of women (12.50%) compared to the control group (36.67%) (*p* = 0.026), suggesting a potential gender disparity in MI risk within this subgroup. Weight status was a key differentiator, with the MI group showing a substantially higher prevalence of overweight and obesity (84.38%), while the majority of the control group had a standard weight (73.33%) (*p* < 0.001). This suggests that increased weight is closely associated with MI risk in individuals with low Lipoprotein (a) levels. Furthermore, a higher percentage of smokers was found in the MI group (68.75%) compared to the control group (43.33%) (*p* = 0.044), indicating that smoking is a significant risk factor for MI in this subgroup. The MI group also had significantly worse cholesterol profiles, including higher total and LDL cholesterol and lower HDL cholesterol levels (all *p* < 0.05), highlighting the role of poor lipid profiles in increasing MI risk among individuals with low Lipoprotein (a).

High Lipoprotein (a) (≥30 mg/dL) (Table 3):

In contrast, among individuals with Lipoprotein (a) levels ≥ 30 mg/dL, the differences between the MI and control groups were less pronounced. There was no significant difference in gender distribution between the two groups (*p* = 0.836), suggesting that sex does not play a major role in MI risk for those with elevated Lipoprotein (a) levels. Unlike the low Lipoprotein (a) group, the weight distribution was similar between the MI and control groups, with no significant differences in the proportions of standard weight, overweight, or obese individuals (*p* = 0.800), indicating that weight may be less influential in MI risk for individuals with high Lipoprotein (a). Smoking rates were comparable in both groups (*p* = 0.895), suggesting no significant difference in smoking status between those who experienced MI and those who did not in this high Lipoprotein (a) subgroup. Although total and LDL cholesterol levels were similar between the MI and control groups, the MI group had significantly lower HDL cholesterol levels (*p* = 0.020), indicating that low HDL may still contribute to MI risk in individuals with elevated Lipoprotein (a) levels.

Multivariate analysis of the risk of acute myocardial infarction in patients with high Lipoprotein (a) levels.

The logistic regression analysis (Table 4) identified critical risk factors for acute myocardial infarction (AMI). Male participants exhibited higher odds of developing AMI (odds ratio [OR] = 2.38; *p* = 0.137); however, this association did not achieve statistical significance (95% confidence interval [CI]: 0.75–7.50). Overweight individuals demonstrated more than a twofold increase in the likelihood of experiencing AMI compared to those with a standard weight (OR = 2.29; *p* = 0.017), indicating a significant elevation in risk. Smoking status was not significantly correlated with AMI in this model (OR = 1.15; *p* = 0.784).

Total cholesterol levels showed a nonsignificant trend toward increased AMI risk (OR = 1.02; *p* = 0.088; 95% CI: 0.99–1.05). Conversely, high-density lipoprotein (HDL) cholesterol levels were inversely associated with AMI risk, exhibiting a protective effect (OR = 0.90; *p* = 0.001). Low-density lipoprotein (LDL) cholesterol levels did not demonstrate a significant association with AMI occurrence (OR = 0.98; *p* = 0.509).

Crucially, elevated Lipoprotein (a) levels greater than 30 mg/dL were significantly associated with increased odds of AMI (OR = 3.01; *p* = 0.042). This finding suggests that high Lipoprotein (a) concentrations are an independent risk factor for acute myocardial infarction.

## 4. Discussion

This study emphasizes the link between elevated Lipoprotein (a) (Lp(a)) levels and an increased risk of acute myocardial infarction (AMI). Through multivariate analysis, it was demonstrated that individuals with Lp(a) concentrations ≥ 30 mg/dL have a threefold increased risk of experiencing AMI [8]. Our results are aligned with major studies in the specialized literature that identify Lp(a) as an important marker for myocardial infarction risk due to its proatherogenic and pro-thrombotic effects [9]. Furthermore, this study reaffirms the protective role of high-density lipoprotein cholesterol (HDL-C) against AMI. Lower HDL-C levels were identified as a significant predictor of AMI, particularly in patients with elevated Lp(a), emphasizing the importance of optimal lipid profile management for mitigating cardiovascular risk [10]. Moreover, the results of this study have significant clinical implications, as Lp(a) may have a greater influence than other risk factors; introducing this biomarker into clinical practice could provide a more accurate estimation of myocardial infarction risk [11].

The analysis of risk factor distribution in the group of patients with AMI compared to the control group revealed a significant association between male sex, nutritional status, smoking status, deteriorated lipid profiles, and elevated Lipoprotein (a) levels with an increased risk of myocardial infarction, both through univariate and multivariate analysis.

Male sex was significantly associated with an increased risk of AMI. The relationship between sex and the risk of acute myocardial infarction has been extensively studied. Men have a higher incidence of myocardial infarction compared to women across various age groups and risk factors [12,13]. Generally, men exhibit higher levels of traditional risk factors such as smoking, high blood pressure, and elevated cholesterol levels, which contribute to their higher risk of AMI. The sex difference in AMI risk decreases with advancing age, but men consistently present a higher risk throughout life [14,15]. In older age groups, the relative risk of AMI in men compared to women decreases but remains significant [15].

Overweight individuals had a significantly higher risk of myocardial infarction in both univariate and multivariate analyses, being a well-known risk factor for AMI [16]. The risk of AMI is higher in overweight and obese individuals, regardless of the presence of metabolic syndrome [17]. A 2014 meta-analysis that included 36,803 participants, of whom 14,883 had an AMI, confirmed that both overweight and obesity significantly increase the incidence of AMI [18]. 

The results suggest that smoking status did not strongly predict the risk of myocardial infarction in this study. This result is surprising, given the established link between smoking and cardiovascular diseases, implying that other factors may play a more significant role in the studied population. It is possible that the sample size or specific characteristics of the participants influenced these results, underscoring the need for further research to clarify this observation globally [19,20]. This study identified that in the group of patients with low levels of Lipoprotein (a), traditional risk factors were significant, while in the group with high levels of Lipoprotein (a), the same risk factors were not significant. This suggests that elevated levels of Lipoprotein (a) represent a strong risk factor that may outweigh the influence of demographic and metabolic factors [21].

The high levels of Lp(a) in young individuals with myocardial infarction suggests a strong association between elevated Lp(a) levels and the risk of myocardial infarction in this group. The higher median Lp(a) level of control patients in the 40–50 years age group is somehow counterintuitive, and it may indicate that elevated Lp(a) is not a predominant risk factor for MI in individuals aged 40–50 years. Alternatively, it could be due to sample variability, selection bias, or confounding factors such as lifestyle, genetic predispositions, or the presence of other comorbidities that were not accounted for. In individuals over 50 years old, the similar levels of Lp(a) in both groups suggest that while elevated Lp(a) may contribute to MI risk in older adults, its impact is less pronounced compared to younger individuals. Other risk factors like hypertension, diabetes, and established atherosclerosis might play a more significant role in this age group.

Another important aspect is the impact of elevated Lp(a) levels on the amount of atheromatous plaque in the vascular system [22]. A relevant study to mention is one in which elevated Lp(a) levels were associated with multivessel coronary artery disease in patients with myocardial infarction. Our findings align with this, further reinforcing the association between Lp(a) levels and advanced coronary pathology, suggesting that higher Lp(a) levels may be linked to a more severe burden of vascular disease [23].

Furthermore, given the major role of Lp(a) in increasing cardiovascular risk, it is essential to explore new therapeutic options aimed at lowering Lp(a) levels and to evaluate their impact on the prevention of myocardial infarction [24,25]. Studies are also needed to better understand the interaction between Lp(a) and other cardiovascular risk factors, such as chronic inflammation and oxidative stress.

The observed distribution of Lp(a) levels across age and weight categories provides insight into the nuanced role Lp(a) may play in myocardial infarction risk. The high Lp(a) levels in young MI patients underscore Lp(a) as a potent risk factor in this age group, potentially due to its genetically determined and less modifiable nature. This association diminishes with age, suggesting that other risk factors may exert a stronger influence on MI risk as individuals grow older. The unexpected elevation of Lp(a) in middle-aged controls compared to MI patients in the same age bracket could reflect lifestyle or genetic factors that mitigate Lp(a)‘s impact in this subgroup. Additionally, the elevated Lp(a) levels in standard-weight MI patients, as opposed to their overweight and obese counterparts, suggest that Lp(a) may independently elevate cardiovascular risk, especially in the absence of other conventional risk factors like obesity. In contrast, the higher Lp(a) levels in obese controls hint at a complex interplay where Lp(a) may not directly correlate with MI risk in individuals with multiple metabolic risk factors, potentially due to the overwhelming impact of conditions like metabolic syndrome or other lipid abnormalities [2].

Building on these findings, this study underscores the pivotal role of Lipoprotein (a) levels in modulating cardiovascular risk profiles. Specifically, the analysis revealed that among patients with low concentrations of Lipoprotein (a), traditional risk factors such as age, sex, smoking status, and metabolic indicators (e.g., cholesterol levels and body mass index) were statistically significant in predicting cardiovascular events. In contrast, in patients with high levels of Lipoprotein (a), these conventional risk factors did not show a statistically significant correlation with cardiovascular outcomes. This indicates that elevated levels of Lipoprotein (a) may independently potentiate cardiovascular risk, overshadowing the influences of demographic and metabolic variables.

Elevated Lipo(a) levels are particularly concerning because they are not easily influenced by lifestyle factors or standard lipid-lowering medications like statins, which makes managing this risk factor more challenging. Genetic mutations in LPA can lead to increased production of apolipoprotein (a), a key component of Lipo(a), which results in increased lipoprotein levels that promote atherogenesis. LDL and total cholesterol levels are modifiable by lipid-lowering treatments and lifestyle interventions. However, an analysis of age distribution relative to Lp(a) levels underscores the stability of Lp(a) as an independent cardiovascular risk factor, particularly among younger patients, irrespective of conventional risk determinants. Thus, lipoprotein (a) emerges as a potentially robust predictor of myocardial infarction risk within the broader population.

Our findings will provide new insights into how Lp(a) contributes to cardiovascular diseases, with a particular focus on MI, and will aid in assessing the potential of targeting Lp(a) as a new therapeutic approach for prevention. The results of this study could lead to more personalized and effective strategies for managing MI risk, especially in patients with elevated Lp(a) levels who may not exhibit other conventional risk indicators.

### Limitations

Our research presents several limitations worth mentioning. First, the relatively small sample size limits the ability to generalize the results to a broader population. Second, conducting the research in a single center may have introduced bias related to the specifics of the studied population. Also, we did not consider all possible confounding factors, such as the use of lipid-lowering medications, which could have influenced the levels of Lp(a) and the risk of myocardial infarction.

In this study, we included both STEMI and NSTEMI patients in a single mixed group. This approach was taken to ensure a sufficient sample size and to investigate the potential commonalities in the relationship between elevated Lipoprotein (a) levels and acute myocardial infarction across different presentations of myocardial infarction. We acknowledge that this choice could limit the specificity of our findings. Future studies might benefit from examining STEMI and NSTEMI patients separately to explore these differences in more detail.

We would like to note that another limitation of the study is the acute-phase reactant nature of Lp(a), with its values potentially influenced during an acute coronary event.

Despite these limitations, our findings provide significant evidence that can guide future research and clinical practice. It is important for subsequent studies to investigate in depth the mechanisms by which Lp(a) contributes to the development of atherosclerosis and myocardial infarction [19]. Additional research, with larger and geographically diverse samples, is necessary to validate these results and apply them to other populations.

## 5. Conclusions

Lipoprotein (a) has been shown to be a significant independent risk factor for acute myocardial infarction (AMI), surpassing the influence of traditional cardiovascular risk factors such as cholesterol levels, smoking, and body weight in patients with elevated Lp(a) levels. Our study highlights the importance of monitoring Lp(a) in cardiovascular risk assessment, offering a more accurate method of identifying high-risk individuals, even in the absence of conventional risk factors.

Incorporating Lp(a) into clinical practice could pave the way for personalized therapeutic strategies aimed at reducing this marker and preventing major cardiovascular events. Future research should focus on exploring the mechanisms through which Lp(a) contributes to atherosclerosis and identifying new therapeutic options for high-risk patients.

## Figures and Tables

**Table 1:** Characteristics of the patients with acute coronary syndrome included in the study. HBP: high blood pressure. HDL: high-density lipoprotein cholesterol. LDL: low-density lipoprotein cholesterol.

Parameter	Control Group (*n* = 42)	Myocardial Infarction Group (*n* = 64)	*p*
	No. (%) Median (IQR)	No. (%) Median (IQR)	
**Sex**			0.132
Women	14 (33.33%)	13 (20.31%)	
Men	28 (66.67%)	51 (79.69%	
**Age**	34 (29–40)	48.5 (43.5–54)	<0.001 *
**BMI**	22.5 (17.5–26.9)	28.5 (24.9–32.2)	<0.001 *
**Overweight**			<0.001 *
Standard weight	27 (64.29%)	17 (26.56%)	
Overweight	10 (23.81%)	22 (34.38%)	
Obesity	5 (11.90%)	25 (39.06%)	
**Smoking status**			0.050 *
Smoker	21 (50.00%)	44 (68.75%)	
Non-smoker	21 (50.00%)	20 (31.25%)	
**Diabetes mellitus**	0 (0.00%)	18 (28.12%)	<0.001 *
**HBP**	0 (0.00%)	43 (67.19%)	<0.001 *
**LVEF (%)**	61 (55–65)	50 (45–55)	
**Blood parameters**			
WBC (/uL)	7.0 (5.5–8.5)	8.9 (7.3–11.8)	<0.001 *
HBG (g/dL)	14.1 (13.2–14.5)	14.2 (13.4–15.2)	0.624
PLT (/uL)	250 (205–315)	243 (216–278)	0.547
UREA (mg/dL)	28 (24–32)	31 (25.5–35.5)	0.013 *
CRE (mg/dL)	0.9 (0.7–1.0)	0.83 (0.75–0.95)	0.038 *
GLU (mg/dL)	91.0 (82–100)	108.5 (94–129)	<0.001 *
TROP (ng/L)	0.7 (0.1–2.5)	3.1 (1.2–10.4)	0.009 *
CK-MB	7.6 (4.8–10.1)	310 (220–475)	<0.001 *
Total cholesterol	172 (162–200)	205 (167.5–235)	0.002 *
LDL cholesterol	100.5 (85–122)	126.3 (90.2–162.1)	0.006 *
HDL cholesterol	50.5 (45.5–56)	39.6 (32.6–45.4)	<0.001 *
**Lipoprotein (a) level**			
<30 mg/dL	30 (71.43%)	32 (50.00%)	0.028 *
≥30 mg/dL	12 (28.57%)	32 (50.00%)	
**Age distribution**			
<40 years	12.9 (8.3–29.3)	126.4 (17.7–180.8)	
40–50 years	42.3 (9.6–89.2)	34.9 (14.9–165.6)	
>50 years	11.9 (2.7–24.9)	14.8 (6.5–70.5)	
**Overweight**			
Standard weight	12.9 (6.7–27)	82.1 (12.7–165.6)	
Overweight	27.1 (8.3–43.9)	19.35 (7.9–142.6)	
Obesity	89.2 (11.9–150.3)	23.9 (11–100.3)	

IQR: interquartile range; *: *p* < 0.05.

**Table 2:** Differences in biological parameters between the control group and myocardial infarction group in patients with Lipoprotein (a) < 30 mg/dL.

Parameter	Lipoprotein (a) < 30 mg/dL		*p*
	Control Group	Myocardial Infarction Group	
	No. (%) Median (IQR)	No. (%) Median (IQR)	
**Sex**			0.026 *
Women	11 (36.67%)	4 (12.50%)	
Men	19 (63.3%)	28 (87.50%)	
**Overweight**			<0.001 *
Standard weight	22 (73.33%)	5 (15.63%)	
Overweight	6 (20.00%)	13 (40.63%)	
Obesity	2 (6.67%)	14 (43.75%)	
**Smoking status**			0.044 *
Smoker	13 (43.33%)	22 (68.75%)	
Non-smoker	17(56.67%)	10 (31.25%)	
**Lipid profile**			
Total cholesterol	167 (155–174)	209 (181–233)	<0.001 *
LDL cholesterol	92.5 (80–126.3)	126 (88–153)	0.020 *
HDL cholesterol	53 (47–56)	36 (30–45)	<0.001 *

IQR: interquartile range; *: *p* < 0.05.

**Table 3:** Differences in biological parameters between the control group and myocardial infarction group in patients with lipoprotein (a) ≥ 30 mg/dL.

Parameter	Lipoprotein (a) ≥ 30 mg/dL		*p*
	Control Group	Myocardial Infarction Group	
	No. (%) Median (IQR)	No. (%) Median (IQR)	
**Sex**			0.836
Women	3 (25.00%)	9 (28.13%)	
Men	9 (75.00%)	23 (71.87%)	
**Overweight**			0.800
Standard weight	5 (41.67%)	12 (37.50%)	
Overweight	4 (33.33%)	9 (28.13%)	
Obesity	3 (25.00%)	11 (34.38%)	
**Smoking status**			0.895
Smoker	8 (66.67%)	22 (68.75%)	
Non-smoker	4(33.33%)	10 (31.25%)	
**Lipid profile**			
Total cholesterol	201 (181–210)	201 (153–171)	0.843
LDL cholesterol	121 (109–135)	125 (98–171)	0.571
HDL cholesterol	47 (44–54)	40 (38–50)	0.020 *

IQR: interquartile range; *: *p* < 0.05.

**Table 4:** Logistic regression analysis of risk factors for acute myocardial infarction.

Risk Factors	Odds Ratio	Std. Err.	z	*p* > |z|	[95% Conf. Interval]	
Lipo(a) > 30 mg/dL	3.01	1.63	2.03	0.042 *	1.03	8.74
HDL-C (mg/dL)	0.90	0.02	−3.24	0.001 *	0.85	0.96
LDL-C (mg/dL)	0.98	0.01	−0.66	0.509	0.95	1.02
Total cholesterol (mg/dL)	1.02	0.01	1.71	0.088	0.99	1.05
Male sex	2.38	1.39	1.49	0.137	0.75	7.50
Overweight status	2.29	0.80	2.38	0.017 *	1.15	4.55
Smoking status	1.15	0.61	0.27	0.784	0.40	3.29
Constant	0.62	1.02	−0.29	0.775	0.02	15.74

*: *p* < 0.05.

## Data Availability

The data are available from the authors upon request.
